# Bicultural Minds: A Cultural Priming Approach to the Self-Bias Effect

**DOI:** 10.3390/bs12020045

**Published:** 2022-02-11

**Authors:** Mengyin Jiang, Jie Sui

**Affiliations:** 1 Academy of Arts and Design, Tsinghua University, Beijing 100084, China; 2 The Future Laboratory, Tsinghua University, Beijing 100084, China; 3 Department of Experimental Psychology, University of Oxford, Oxford OX2 6GG, UK; 4 School of Psychology, University of Aberdeen, Aberdeen AB24 3FX, UK; jie.sui@abdn.ac.uk

**Keywords:** self-bias, perceptual matching, priming, culture, independent self-construal, interdependent self-construal

## Abstract

Recent research has discovered a robust bias towards the processing of self-relevant information in perceptual matching. Self-associated stimuli are processed faster and more accurately than other-associated stimuli. Priming of independent or interdependent self-construal can dynamically modulate self-biases in high-level cognitive tasks. This study explored whether priming of independent/interdependent mindsets can modulate the self-bias effect in perceptual matching. In two experiments, British participants performed a priming task (Experiment 1 using a word-search task—an implicit priming approach, Experiment 2 with a reflective thinking task—an explicit priming method) immediately followed by a perceptual matching task, where they first learned to associate geometric shapes with labels (e.g., circle is you, square is friend, triangle is stranger) and then made judgments on whether shape-label pairs displayed on-screen were the correct associations or not. The analysis in Experiment 1 revealed that priming the interdependent self-construal led to a reduced self-bias effect in perceptual matching in participants who had low bias compared to those with high bias in the neutral/non-priming condition. In contrast, priming the independent self-construal did not modulate the self-bias in perceptual matching. The effects were replicated in Experiment 2. The results indicate that the self is a dynamic concept that can modulate perceptual processing by accessing different cultural contexts.

## 1. Introduction

People often prioritize information related to the self [1,2]. This prioritization of self-related information processing (i.e., faster responses and greater accuracy) is known as the self-bias effect and has been observed in many cognitive studies [3,4]. For instance, dichotic listening studies that presented different messages to each ear found that participants were able to report instances of self-name presented in the ignored ear, even when completely oblivious to other information presented in that same ear [5,6]. Information encoded in self-reference also has a significant advantage over information encoded in reference to others in both memory recognition and free recall [7,8,9].

A perceptual matching paradigm developed by Sui and colleagues [10] further confirmed the robustness of self-bias while controlling the effects of stimulus familiarity and complexity. In this task, participants first associated a neutral geometric shape (i.e., triangle, square, or circle) with a label (i.e., self, friend, or stranger), e.g., triangle is you, square is your best friend, and circle is a stranger. Then, combinations of a shape with a label were presented on-screen and participants had to make judgments on whether the presented shape and label pair were the correct association or not. Responses were faster and more accurate to the self-associations than to friend and stranger associations. The self-bias effect in perceptual matching has consistently been reported in a range of cognitive tasks [11,12,13], and it is difficult to avoid [14] even without the presence of familiar labels [15,16]. Although the strength of the self-bias may vary across tasks and between individuals [17], it remains unknown if self-bias in perceptual matching can be modulated by independent and interdependent self-construals.

The self is a dynamic construal shaped by socio-cultural experiences [18,19]. A key differentiation between independent and interdependent self-construals is the perception of the self in relation to others [20]. Independent self-construal tends to emphasize independence, and the self is construed as an autonomous entity reflecting the goals of the individual. In contrast, interdependent self-construal tends to emphasize interdependence, where the self is construed as socially embedded among family and friends [21]. This difference between independent and interdependent self-construals has been observed in many studies. For example, Sparks and colleagues found differences in self-bias due to independent and interdependent self-construals [22]. While participants with independent self-construal demonstrated a self-advantage in recognition memory accuracy for self-owned objects, no self-advantage was found in participants with interdependent self-construal. Additionally, when the task required more attention, participants with interdependent self-construal showed higher recognition accuracy for mother-owned (a significant other) rather than self-owned objects. This demonstrates that for an interdependent mind, significant others such as family or friends are particularly important and can have great impact on one’s cognition.

Although the orientations of independence and interdependence were previously portrayed as opposing concepts [23], both self-construal styles co-exist within the same individual [24,25]. In fact, it was found that people with more prominent independent self-construal styles were not necessarily less interdependent than people with more prominent interdependent self-construal styles [19]. It was proposed that the two self-construal styles are not mutually exclusive but two parallel dimensions with one more easily accessible than the other [26]. Thus, it is possible to shift between independent and interdependent mindsets through priming within the same individual, which subsequently affects perceptual judgments and memory [27,28].

The dynamic shift between independent and interdependent self-construal can be achieved through relatively simple tasks. Brewer and Gardner [29] primed Caucasian American participants with word search tasks [30,31]. After circling interdependent pronouns such as “we”, participant responses showed significantly more interdependent self-descriptions that described group membership. Kuhnen and Oyserman [32] also showed that word search tasks with interdependent pronouns (we, our, us) induced context-sensitive processing, while independent pronouns (I, my, me) induced context-insensitive processing. The pronoun word search task has demonstrated successful priming in multiple languages [33,34], suggesting that it is a stable effect on the shift between independent and interdependent mindsets. Writing essays on independent or interdependent themes can also achieve successful priming. The Similarities and Differences with Family and Friends (SDFF) task primed participants with essay questions that either emphasized personal expectations (independent priming) or family expectations (interdependent priming) [35,36,37]. After priming, participants demonstrated values that corresponded with the priming condition—more independent values after independent priming, and more interdependent values after interdependent priming.

These independent and interdependent priming approaches are most effective when they are incongruent with the participants’ chronic or default sociocultural mindsets [20,38]. Default mindset refers to the prominent frame of mind that the participants have chronic access and experience to. In other words, independence is the default mindset for most Westerners who identify the self as an autonomous individual, while interdependence is the default mindset for most East Asians who recognize the self through connections to family and friends. Thus, the British participants recruited in this study would be expected to show the most prominent results after interdependent (vs. independent) priming.

Following these lines of research, the current study was designed to explore three main questions. The first was whether incongruent (i.e., interdependent) priming could successfully modulate the self-bias effect in perceptual matching in British participants. Secondly, evidence on individual differences in the perception of the self in relation to others [39,40] indicates that self-bias in perceptual matching can be modulated, but only in individuals with weaker self-bias [41]; we therefore also explored whether the modulating effect would be most prominent in British participants with low self-biases as measured by the neutral/non-priming condition. The third question was whether the same results could be replicated with both implicit and explicit priming methods, which has previously been studied in isolation. Two experiments were conducted: Experiment 1 examined the effects of implicit priming on self-bias through a word-search task [42], while Experiment 2 examined the effects of explicit priming on self-bias through an SDFF task [35,36]. Using a within-subject design, each participant was primed with three conditions—neutral/no-priming, independent priming, and interdependent priming. Following each priming condition, the perceptual matching task was performed. The perceptual matching paradigm has been used extensively to research a broad range of topics such as reward and self-related processing [43], self and emotion [44], and ingroup vs. outgroup biases [45], and has been demonstrated to be an especially suitable tool for making comparisons. Thus, the current study employed the perceptual matching task to compare self-biases after each priming condition. 

## 2. Materials and Methods

### 2.1. Experiment 1: Implicit Priming

#### 2.1.1. Participants

Thirty-six healthy volunteers (10 male, 26 female, 18 to 35 years of age, mean age ± standard deviation = 22.22 ± 4.51) took part in this study. All participants were Caucasian British undergraduate and postgraduate students, right-handed, and had normal or corrected-to-normal vision. Informed consent was obtained from all participants prior to the experiment. The procedure used in this experiment was ethically approved by the University of Oxford Central University Research Ethics Committee.

#### 2.1.2. Stimuli and Materials

Participants learned to associate a shape with a label and were asked to make judgments on whether the shape and label pair shown on the screen matched the associations previously learnt. For all participants, a white fixation cross was presented at the center of the screen at 0.8 × 0.8 degrees of visual angle. One of three geometric shapes (triangle, square, or circle) was presented above the fixation cross at 3.8 × 3.8 degrees of visual angle, and one of three labels (you, friend, or stranger) was presented below the fixation cross at 3.1/3.6 × 1.6 degrees of visual angle. The association of shapes with labels was counterbalanced across participants. The distance between the shape/word to the fixation cross was 3.5 degrees of visual angle. Stimuli were presented in a grey background on a 23-inch monitor (1920 × 1400 at 60 Hz). The program was run on a PC using E-prime software (version 2.0). All stimuli were consistent with those used in the study by Sui and colleagues [10].

Priming materials can be found in Supplementary Materials.

#### 2.1.3. Procedure

**Figure 1:**
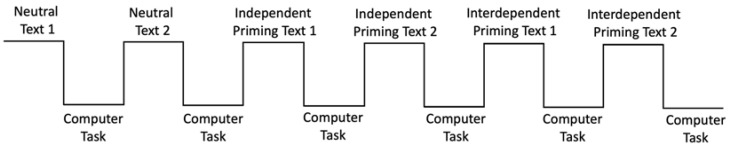
A schematic of the experiment procedure. The order of the independent and interdependent priming was counterbalanced between subjects.

Each participant alternated between the word search task and the computer-based matching task six times (see Figure 1). The six word search tasks consisted of two neutral texts (texts with no pronouns), two independent priming texts, and two interdependent priming texts. Each priming text described a trip to a tourist destination either not using pronouns or using pronouns such as “I, me, myself” or “we, us, our”. Participants were instructed to read each text and circle either target words or pronouns. In the neutral texts, participants were instructed to circle all instances of neutral words (e.g., park, area, pyramid, giza, sphinx). The number of instances of target words was the same for both texts. In the independent and interdependent priming texts, participants were asked to circle all the pronouns (e.g., I, my, me, mine in the independent priming text and we, our, ours, us in the interdependent priming text). Participants received verbal instructions to read each text three times to make sure all the relevant words were circled. Following each priming text, participants subsequently performed a shape-label association task on the computer (see Figure 1).

**Figure 2:**
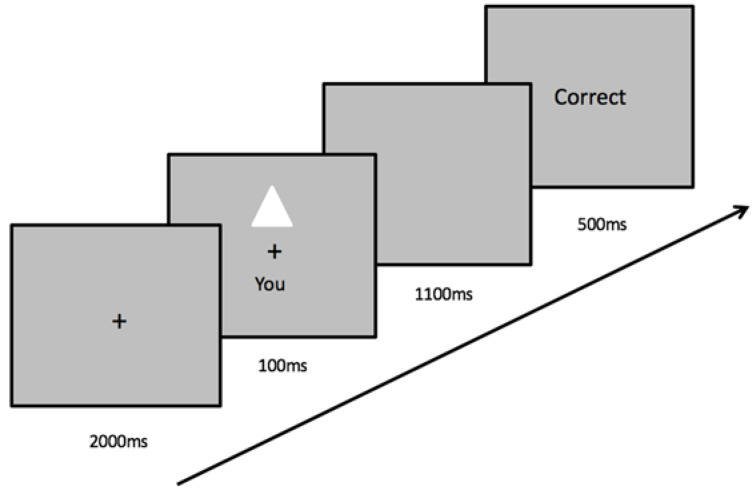
A trial procedure and an example of stimuli in the matching task. Participants responded by making judgments on whether the shape-label pair displayed onscreen is the correct association or not.

Each perceptual matching task consisted of a training stage and a matching stage. In the training stage, participants learned to associate a shape with a label. For example, the triangle represents your best friend, the square represents you, and the circle represents a stranger. These learned association pairs remained the same throughout the six computer tasks for each participant. At this stage, the shapes were not presented on the screen. In the matching stage, the participants were asked to place both index fingers on one of two keys on the keyboard and made judgments about the shape and label pair shown on the screen. At the beginning of each trial, a fixation cross was presented at the center of the screen for 2000 ms, followed by a pair of shape and label above and below the fixation cross for 100 ms. The shape and label pair either matched the associations previously learned in the training stage or was a recombination of a shape with a label randomly generated by the computer task. Next, the screen remained blank for 1100 ms, during which time the participants were expected to respond by pressing one of the two buttons as quickly and accurately as possible. Following a response, feedback (green “Correct” or red “Incorrect”) was given on the screen for 500 ms at the end of each trial. If no response was given within the 1100 ms timeframe, the feedback “Too Slow!” was displayed in yellow to prompt responses (see Figure 2). Feedback on overall accuracy was provided at the end of each block. The participants performed nine practice trials and three blocks of 60 trials following each priming condition.

Each participant performed two cycles of each priming consecutively. The experiment always started with the neutral task to establish the participants’ baseline for the self-biases. Then, the participant either performed two cycles of the independent (circle pronouns such as ‘I’) or interdependent tasks (circle pronouns such as ‘we’) followed by two interdependent or independent tasks (see Figure 1). The order of independent and interdependent priming tasks was administered in a counterbalanced order across participants.

#### 2.1.4. Experimental Design and Data Analyses

Consistent with previous studies, the self-bias effect was quantified by calculating the response difference between self and other (friend or stranger) in mean correct reaction times (RTs) and d-prime [10,46].

Based on the design of the current experiment, neutral/no-priming and priming conditions were analyzed separately. The self-bias relative to stranger (e.g., stranger RT—self RT) in the neutral condition was calculated as a baseline to establish the magnitude of one’s self-bias without interference from priming. The rationale was that a stranger is the least self-related person available. Meanwhile, self-bias relative to friend (e.g., friend RT—self RT) from the priming conditions was calculated and used as an index for the modulation of the self-bias effect. This was due to the idea that the key differentiation between independent and interdependent self-construal is the perception of the self in relation to significant others [18]. Therefore, a reduced self-bias relative to friend would indicate a shift toward an interdependent self-construal (i.e., the self is socially embedded among family and friends), whereas an increased self-bias relative to friend would indicate an independent self-construal (i.e., the self is an autonomous individual).

Data analyses were performed and reported in two sections—RTs in match trials and d-prime. In the match trials, RTs from the neutral condition and priming conditions were analyzed separately. In the neutral condition, an ANOVA was performed with one within-subjects variable—shape-label association. As a separate dataset, the priming conditions were analyzed with 2 within-subject variables—priming condition (independent or interdependent) and shape-label association (self, friend, stranger)—and a between-subject variable—bias group (low self-bias or high self-bias). D-prime scores for the neutral and priming conditions were also analyzed separately using the same method. Bonferroni correction was applied for multiple comparisons [47] in paired-sample *t*-tests.

For each participant in each condition, mean RTs for the correct responses were calculated. Response times shorter than 200 ms were excluded from the analysis, as were RTs falling more than 3 standard deviations outside the mean for that condition. This eliminated less than 1% of the trials overall. Data from four participants were excluded from the analysis—two participants were excluded due to low accuracy that was close to chance level, and two other participants on the boundaries of the two bias groups were excluded in order to maximize the bias group effects.

Initial analyses on the mismatch trials did not reveal any significant findings and thus are reported in Supplementary Materials.

### 2.2. Experiment 2: Explicit Priming

#### 2.2.1. Participants

Thirty-six healthy volunteers (11 male, 25 female, 18 to 32 years of age, mean age ± standard deviation = 21.61 ± 3.51) took part in this study. All participants were Caucasian British undergraduate and postgraduate students, right-handed, and had normal or corrected-to-normal vision. Informed consent was obtained from all participants prior to the experiment. The procedure used in this experiment was ethically approved by the University of Oxford Central University Research Ethics Committee.

#### 2.2.2. Stimuli and Materials

Stimuli used in this study were the same as in the Implicit Priming experiment.

#### 2.2.3. Procedure

**Figure 3:**
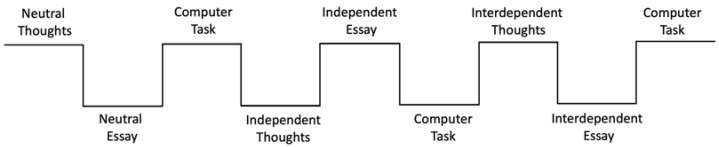
A schematic of the experiment procedure. The order of all three conditions was counterbalanced between subjects.

Each participant alternated between an essay-writing priming task and the computer-based perceptual matching task three times (see Figure 3). The essay-writing priming task was a modification of the SDFF task used in the study by Chiao and colleagues [35]. Participants were asked to think about a question for two minutes, and then to write a short essay in response to another question for eight minutes. In the neutral condition, participants were given the instructions “*For the next two minutes, please think of what items are in your bedroom*”, and then to write a short essay in response to the question “*What do you have in your bedroom?*”. For the independent priming conditions, participants were asked to think about “*What makes you unique from your friends and family?*” and to write an essay in response to “*What do you expect yourself to do?*”. For the interdependent priming condition, participants were asked to think about “*What you have in common with your friends and family*” and to write about “*What do your friends and family expect you to do?*”. Participants were instructed to write as much as they could and to be as detailed as possible for the essays. Following each priming task, participants performed a perceptual matching task on the computer (see Figure 3).

The perceptual matching task was the same as the one used in the implicit priming experiment. To keep the number of trials the same between the two experiments, participants performed six blocks of 60 trials instead of three blocks after each priming task. In Experiment 1, the idea was to establish one’s baseline of self-biases first, and thus only the priming conditions were counterbalanced. However, it was later realized that this may cause a learning effect in the results. To avoid this, all three priming conditions were administered in a counterbalanced order in Experiment 2.

#### 2.2.4. Experimental Design and Data Analyses

Data analyses were performed and organized in the same way as in Experiment 1. Response times shorter than 200 ms and RTs falling more than 3 standard deviations outside the mean for that condition were excluded from the analysis, which eliminated less than 1% of the overall trials. Again, self-bias relative to stranger (stranger RTs—self RTs) from the neutral condition was used to divide participants into low or high-bias groups. Data from four participants on the boundaries of the two bias groups were excluded to maximize the bias group effects.

Bonferroni correction [47] was applied to paired-sample *t*-tests.

## 3. Results

### 3.1. Experiment 1: Implicit Priming

#### 3.1.1. Neutral Condition

##### RTs in Match Trials

A repeated measures ANOVA revealed a significant main effect of shape-label association, *F*(2, 62) = 49.03, *p* < 0.001, η^2^ = 0.61 (see Table 1). Pairwise comparisons showed that RTs for the self-association were significantly faster than both friend (*p* < 0.001) and stranger associations (*p* < 0.001), showing the effect of self-bias. The RTs for friend association were also significantly faster than for stranger association (*p* < 0.01), suggesting a bias towards stimuli that were more self-related.

Self-bias relative to stranger was calculated (stranger RT—self RT) to establish the magnitude of self-bias for each participant, which was used in a median split to divide participants into low and high-bias groups in the priming conditions.

##### D-Prime

Using the Green and Swets formula [48], d-prime was calculated for each participant to determine participants’ sensitivity to correct and incorrect associations across both match and mismatch shape-based associations.

The data from the neutral condition were analyzed using the shape-label association (self, friend, or stranger) as the within-subjects variable. A repeated measures ANOVA revealed a significant main effect of shape-label association, *F*(2, 62) = 9.69, *p* < 0.001, η^2^ = 0.24 (see Table 2). Pairwise comparisons showed that d-prime for the self-association was significantly higher than for friend (*p* < 0.01) and stranger associations (*p* < 0.01). D-prime for the friend association was not significantly different from the stranger association (*p* = 1.00).

#### 3.1.2. Priming Conditions: Interdependent Priming Reduces Self-Bias Relative to Friend in People with Low Bias

##### RTs in Match Trials

Due to individual differences in the magnitude of self-bias established in the neutral condition, low- and high-bias groups were defined and applied to the analyses below. The RTs from the independent and interdependent conditions were analyzed with a mixed design ANOVA, using two within-subjects variables—priming condition (independent or interdependent) and shape-label association (self, friend, or stranger)—and one between-subjects variable—bias group (low or high bias). The analysis revealed a significant main effect of shape-label association, *F*(2, 60) = 36.78, *p* < 0.001, η^2^ = 0.55, but not priming, *F*(1, 30) = 1.54, *p* = 0.23. Responses to the self-association were significantly faster than to both friend (*p* < 0.001) and stranger associations (*p* < 0.001), though no significant differences were found between friend and stranger associations (*p* = 0.42) (see Table 1). A significant interaction was found between shape-label association and bias groups, *F*(2, 60) = 3.81, *p* < 0.05, η^2^ = 0.11, but not between priming and bias group, *F*(1, 30) = 0.00, *p* = 0.97, nor between priming and shape-label association, *F*(2, 60) = 0.30, *p* = 0.74 (see Table 1).

Most importantly, the effects were qualified by a significant three-way interaction between priming condition, shape-label association, and bias group, *F*(2, 60) = 3.47, *p* < 0.05, η^2^ = 0.10.

**Figure 4:**
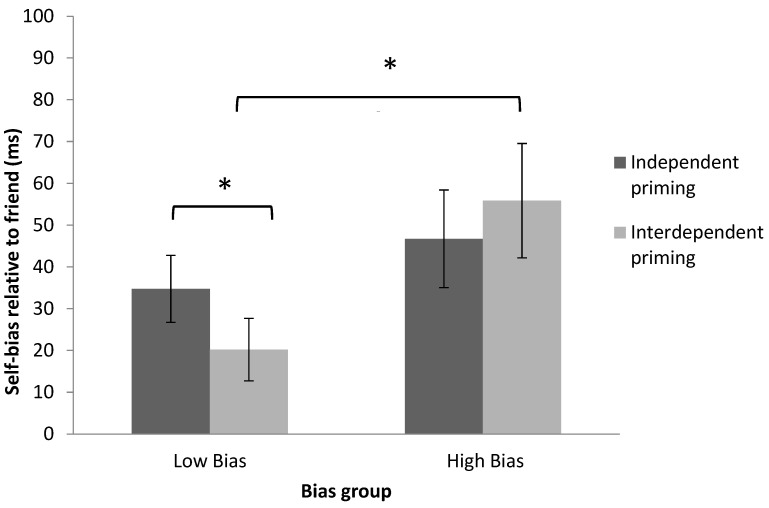
Decomposition of the significant interaction between priming, shape-label association and bias group using the self-bias relative to friend (calculated friend RT—self RT) in Experiment 1. Mixed design ANOVA revealed a significant interaction between the priming and bias group, and the paired-sample *t*-test revealed a significant difference of priming in the low-bias group. Error bars represent one standard errors. Significant results are marked with “*”.

To decompose the three-way interaction between priming, shape-label association, and bias group, a mixed design ANOVA on the self-bias relative to friend (friend RT—self RT) after independent and interdependent priming was performed. This revealed a significant interaction between priming condition (independent or interdependent) and bias group (low or high bias), *F*(1, 30) = 4.12, *p* = 0.05, η^2^ = 0.12 (see Table 1 and Figure 4).

Paired-sample *t*-tests showed that interdependent priming reduced the self-bias relative to friend in participants in the low-bias group compared to independent priming, *t*(15) = 2.24, *p* < 0.05, *dz* = 0.50. However, this difference was not observed in participants in the high-bias group after priming (interdependent vs. independent priming), *t*(15) = −0.94, *p* = 0.36, *dz* = 0.18 (see Figure 4). This indicates that individuals in the low-bias group were more likely to be influenced by contextual cues than those in the high-bias group.

Independent-sample *t*-tests on the self-bias relative to friend revealed that the low-bias group showed significantly lower self-bias relative to friend than the high-bias group after interdependent priming, *t*(30) = −2.36, *p* < 0.05, *dz* = 0.78 (see Figure 4), but not after independent priming, *t*(30) = −0.87, *p* = 0.39, *dz* = 0.30. This demonstrates that interdependent priming successfully decreased differences between self and friend associations among participants in the low-bias group. These effects were consistent with the pattern of results from previous research [20].

##### D-Prime

A mixed design ANOVA was used to analyze the data from the independent and interdependent priming conditions. The two within-subjects variables were priming condition and shape-label association, with bias group as a between-subjects factor. The analysis showed a significant main effect of shape-label association, *F*(2, 60)= 10.82, *p* < 0.01, η^2^ = 0.27. D-prime score for the self-association was significantly higher than friend (*p* < 0.01), and stranger associations (*p* < 0.01), but not significantly different between friend and stranger associations (*p* = 0.96). No main effect of priming was found, *F*(1, 30) = 0.10, *p* = 0.76. A significant interaction was found between shape-label association and bias group, *F*(2, 60) = 5.59, *p* < 0.05, η^2^ = 0.16 (see Table 2). Across priming conditions, the low-bias group showed higher d-prime score for the self-association than friend association, *t*(15) = 2.85, *p* < 0.05, *dz* = 0.61, but not stranger association, *t*(15) = 0.83, *p* = 0.42, *dz* = 0.09, and d-prime score for the stranger association was significantly higher than for the friend association, *t*(15) = −2.97, *p* < 0.05, *dz* = 0.53. The high-bias group, on the other hand, showed higher d-prime score for the self-association than for both friend, *t*(15) = 2.94, *p* < 0.05, *dz* = 0.35, and stranger associations, *t*(15) = 4.46, *p* < 0.001, *dz* = 0.57, but friend and stranger associations were not significantly different, *t*(15) = 1.44, *p* = 0.17, *dz* = 0.20. No interactions were found between priming and bias group, *F*(1, 30) = 1.08, *p* = 0.31, or between priming and shape-label association, *F*(2, 60) = 1.08, *p* = 0.35. No three-way interaction was found between priming, shape-label association and bias group, *F*(2, 60) = 0.15, *p* = 0.86.

### 3.2. Experiment 2: Explicit Priming

#### 3.2.1. Neutral Condition

##### RTs in Match Trials

A repeated measures ANOVA revealed a significant main effect of shape-label association, *F*(2, 62) = 38.84, *p* < 0.001, η^2^ = 0.56 (see Table 3). The RTs for the self-associations were significantly faster than for friend (*p* < 0.001) and stranger associations (*p* < 0.001). However, RTs for the friend association was not significantly different to those of the stranger association (*p* = 0.22).

Consistent with Experiment 1, the self-bias relative to stranger was also calculated to establish the magnitude of self-bias for each participant, which was used to divide participants into low and high-bias groups (using a median split) for data analyses under priming conditions.

##### D-Prime

D-prime scores from the neutral condition were analyzed with shape-label association (self, friend, or stranger) as the within-subjects variable. A repeated measures ANOVA revealed a significant main effect of shape-label association, *F*(2, 62) = 5.37, *p* < 0.01, η^2^ = 0.15 (see Table 4). Pairwise comparisons showed that d-prime score for the self-association were significantly higher than for friend associations (*p* < 0.01) but not for stranger associations (*p* = 0.20). D-prime scores for friend and stranger associations were not significantly different (*p* = 0.70).

#### 3.2.2. Priming Condition: Interdependent Priming Reduces Self-Bias Relative to Friend in People with Low Bias

##### RTs in Match Trials

In line with the analyses performed in Experiment 1, data from the independent and interdependent priming conditions were analyzed together using a mixed design ANOVA. This included priming (independent or interdependent) and shape-label association (friend, self, or stranger) as the two within-subjects variables, and bias group (low or high bias) as the between-subjects variable (see Table 3). The analysis revealed a significant main effect of shape-label association, *F*(2, 60) = 67.62, *p* < 0.001, η^2^ = 0.69. The RTs for the self-association were significant faster than friend (*p* < 0.001) and stranger associations (*p* < 0.001). Friend association also elicited significantly faster RTs than stranger association (*p* < 0.001). No main effect of priming was found, *F*(1, 30) = 0.11, *p* = 0.74. A significant interaction was found between shape-label association and bias groups, *F*(2, 60) = 9.50, *p* < 0.001, η^2^ = 0.24 (see Table 3). No interactions were found between priming and bias group, *F*(1, 30) = 0.00, p = 0.98, or between priming and shape-label association, *F*(2, 60) = 0.64, *p* = 0.53. No significant three-way interaction was found between priming condition, shape-label association, and bias group, *F*(2, 60) = 0.96, *p* = 0.39.

**Figure 5:**
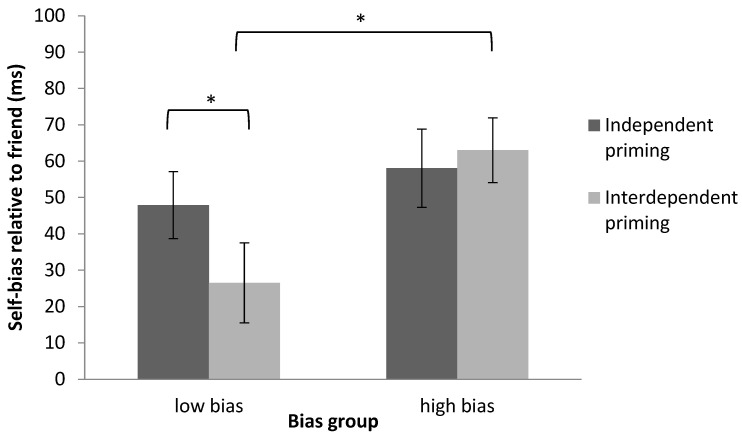
Mixed design ANOVA revealed a significant interaction between shape-label association and bias group in Experiment 2. A paired-sample *t*-test revealed a significant difference of priming in the low-bias group. Significant results are marked with “*”.

Although no significant three-way interaction was found between priming, shape-label association, and bias group, given the results from Experiment 1, the effects of priming were examined using a priori *t*-tests. A paired-sample *t*-test revealed that participants in the low-bias group demonstrated significantly lower self-bias relative to friend after interdependent priming than after independent priming, *t*(15) = 2.26, *p* < 0.05, *dz* = 0.54 (see Figure 5). The effect was not observed in participants in the high-bias group after priming, *t*(15) = −0.42, *p* = 0.68, *dz* = 0.13.

An independent-sample *t*-test on the self-bias relative to friend revealed that after interdependent priming, the low-bias group showed significantly smaller bias than the high-bias group, *t*(30) = −2.94, *p* < 0.01, *dz* = 0.94 (see Figure 5). However, after independent priming, no difference between the low-bias group and the high-bias group was found, *t*(30) = −0.74, *p* = 0.47, *dz* = 0.26. Again, interdependent priming successfully modulated the self-bias relative to friend in participants with low bias. These results replicated the findings from Experiment 1.

##### D-Prime

A mixed design ANOVA was performed using priming condition (independent or interdependent) and shape association (self, friend, or stranger) as the two within-subjects variables, and bias group (low or high bias) as a between-subjects factor. The analysis revealed a significant main effect of shape-label association, *F*(2, 60) = 16.18, *p* < 0.001, η^2^ = 0.35 (see Table 4). Pairwise comparisons showed that d-prime score for the self-association was significantly higher than for friend (*p* < 0.001) and stranger associations (*p* < 0.001). D-prime scores for friend association were not significantly different from those for stranger association (*p* = 0.37). No priming effect was found, *F*(1, 30) = 0.10, *p* = 0.76. No significant interactions were found between priming and bias group, *F*(1, 30) = 0.75, *p* = 0.39, between shape-label association and bias group, *F*(2, 60) = 0.28, *p* = 0.76, or between priming and shape-label association, *F*(2, 60) = 0.34, *p* = 0.71. There was no significant three-way interaction between priming, shape-label association, and bias group, *F*(2, 60) = 0.36, *p* = 0.70.

## 4. Discussion

### 4.1. Experiment 1 Discussion

Experiment 1 investigated the effects of priming on self-bias in British participants using an implicit priming method. The results revealed that incongruent priming (i.e., interdependent priming)—as opposed to congruent (i.e., independent) priming—successfully reduced self-bias relative to friend in participants with low bias. However, this reduced self-bias relative to friend was only observed in participants with low bias and not in those with high bias. This can be explained by individual differences in the magnitude of the self-bias effect and by the fact that some people can shift between independent and interdependent self-construal styles more easily than others. Ellis and Boyce [49] explained that neurobiological variation exists in sensitivity to contextual cues, which determines individual differences in one’s openness to environmental influences. Similarly, people with lower or weaker bias may be more sensitive and susceptible to contextual cues than others.

D-prime scores showed a robust self-bias effect in the significantly higher sensitivity to self-association than to friend and stranger associations. This was found in both the neutral condition and the two priming conditions. Interestingly, across the priming conditions, participants demonstrated different degrees of sensitivity to friend association between the low- and high-bias groups. In the low-bias group, sensitivity for friend was lower than sensitivity for self and stranger. This may be explained by sensitivity to the two extreme opposites of personal perception (self vs. stranger). In contrast, the high-bias group was more sensitive to self-related stimuli than other stimuli, which was consistent with the idea that some people may demonstrate stronger self-bias than others.

A limitation for this experiment was that the neutral condition always took place at the beginning of the experiment and only the order of the independent and interdependent priming was counterbalanced. This may have resulted in a learning effect that only took place in the neutral condition. Ideally, the order of all three conditions should be counterbalanced to avoid any confounding variables. This was taken into consideration in Experiment 2, where all three conditions were counterbalanced.

### 4.2. Experiment 2 Discussion

Experiment 2 employed an explicit priming method to examine the effect of incongruent priming on self-bias. Findings from this experiment replicated the results from Experiment 1, showing a reduced self-bias relative to friend after interdependent priming in people with low bias. Moreover, the RT differences between self and friend were significantly reduced after interdependent priming in the low-bias group compared with those in the high-bias group. The low- and high-bias groups reflected individual differences in the magnitude of the self-bias effect, which suggested that some people were more receptive to contextual cues than others. As a result, interdependent priming was more successful at facilitating the shift from one cultural framework to another in British participants with low-bias.

D-prime scores for the self-association consistently showed greater sensitivity than other associations. This was observed in the neutral condition, as well as across independent and interdependent priming conditions, demonstrating a robust self-bias effect despite contextual cues.

One limitation for this experiment was the essay theme for the neutral condition. Given the within-subjects design of this experiment, it was important that all three conditions were carried out in the same procedure. However, the priming method adapted from the study by Chiao and colleagues [35] only consisted of independent and interdependent priming. Thus, an essay theme was devised to shift one’s focus from independent or interdependent experiences. It was decided that objects in one’s bedroom would be more neutral in the sense that personal items that carry sentimental value may act as a reminder of one’s self-construal style and default cultural experiences. This would help establish a baseline for the participant’s default self-biases. Nevertheless, the essay question has not been tested before and it is unclear whether it was adequate as a neutral condition. Despite this, a priming effect was found in the low-bias group based on the neutral condition, suggesting that the essay question devised for the neutral condition successfully identified low- and high-biased individuals.

### 4.3. General Discussions

This exploratory study aimed to examine three aspects of self-bias through priming: the effect of incongruent priming on the modulation of self-bias within monocultural individuals; the impact of individual differences on the self-bias effect as a result of priming; and replication of the findings using both implicit and explicit priming methods. First, although both experiments revealed a weakened self-bias relative to friend after interdependent priming, this effect only occurred in participants with low bias and not in participants with high bias. In comparison, no significant differences were found after independent priming. Second, the consistency of the results between Experiment 1 and 2 demonstrated that both implicit and explicit cues were equally effective in modulating self-bias in participants with low biases. These results suggested that individual differences exist in the magnitude of the self-bias effect and that people with low self-bias may be more sensitive to contextual cues than others. Alternatively, it could be explained that situational primes such as word-search and SDFF tasks may be a weak simulation of real-life contexts and thus have difficulty eliciting the same responses from all participants. As a result, those who demonstrate larger magnitudes of self-bias remained unconvinced by the contextual primes. Third, both implicit and explicit interdependent priming consistently reduced self-bias over friend in people with low bias, suggesting that the modulation of self-bias is a stable effect that can be replicated and achieved through independent/interdependent priming.

In both experiments, the robustness of the self-bias effect persisted despite bias groups and priming conditions. Though interdependent priming had a modulating effect on the self-bias relative to friend in people with low bias, the participants continued to show an advantage in processing self-related stimulus than friend and stranger stimuli. This result indicated that the self-bias effect in perceptual matching is robust and that self-related information continues to receive prioritized processing over other-related information even in situations that encourage an interdependent mindset. This is consistent with the notion that the self is a special concept that benefits from a unique processing mechanism in the mind and brain [50,51,52].

Our findings align with previous research that different self-construal styles can be accessed through independent and interdependent priming [53,54] and that social context modulates the self-bias effect in monocultural participants [27]. Results from both experiments found that interdependent priming exclusively reduced self-bias in participants with low bias and not in those with high bias. Although the existence of individual differences is well known and respected in psychology, cognitive psychology research often involves population averages rather than individuals [55]. However, one’s interpretation and expectation for stimuli are influenced by many factors such as personal experiences, beliefs, individual personality and identity [56,57]. Thus, it is important to consider the weight of individual differences in studies that focus on individual interpretations of stimuli. The current study has only begun to uncover the possibility of low and high self-biases, and future studies are needed to explore the extent of the impact that individual differences may have on self-related processing.

This study contributes to existing research in three ways. First, most previous priming studies only used one priming method (implicit or explicit) [53,58] and very few have compared implicit and explicit priming. The current study used both priming methods to examine whether one was more effective than the other, which provides a more comprehensive perspective on priming. Second, most previous priming research recruited Asian Americans [59] or Hong Kong Chinese participants [60,61]; both are known to have been extensively exposed to independent and interdependent values. Few studies have examined the shift between independent and interdependent mindsets in individuals within the same culture. The current study provides evidence that independent and interdependent self-construals exist not only in bicultural individuals but also in monocultural individuals. Third, although the field of psychology recognizes individual differences [55], this study directly provides evidence for the impact of individual differences on human cognition and behavior.

One limitation of this study (which applies to Experiment 1 and 2) is that it is unclear how long priming effects last. Previous research suggests that priming may last anywhere from a trial to 200 trials [62,63], and thus carryover effects from the previous prime may dampen the effects of a subsequent prime. Another limitation is that the participants from this study were drawn from university students who may have a higher level of education and come from a higher socioeconomic background than the general population. Moreover, the participants were all from the same cultural background—British. Thus, the generalizability of the findings may be limited due to different socio-cultural values [64], and one must be cautious when drawing conclusions.

Future studies may consider the investigation of the neural responses of the shift between self-construal styles through cultural priming. Previous findings indicated enlarged P1 amplitudes to local than global targets after independent priming and reduced P1 amplitudes to local than global targets after interdependent priming in the occipital region [65]. Incorporating event-related brain potentials (ERP) into the perceptual matching task could potentially reveal more interesting changes in the electrophysiological responses to self-related cues after priming. Moreover, it would further the current understanding of how the brain processes self- and other-relevant information, which subsequently would provide insight on why humans behave differently in various contexts.

## 5. Conclusions

The current study aimed to understand the impact of environmental contexts on the self-bias effect. The self is a complex and dynamic concept that is constantly changing in response to the environment. Although independent self-construal is considered the default mindset for British participants, contextual cues can manipulate the way self-relevant information is processed by accessing the interdependent self. The current results have demonstrated that people adapt to specific situations by alternating between independent and interdependent self-construal styles [66] and that the self-bias effect can be modulated by the environment through both implicit and explicit cues. Understanding the circumstances that motivate people to shift between independent and interdependent mindsets may provide valuable insights on how people behave in response to real-world situations, especially in contexts where group decisions or benefits to the group are in question.

## Figures and Tables

**Table 1:** Mean RTs (ms) and SDs (in brackets) for match trials as a function of association, priming, and bias group in Experiment 1.

	Neutral	Independent Priming		Interdependent Priming	
Association		Low Bias	High Bias	Low Bias	High Bias
Self	633 (68)	625 (64)	600 (68)	624 (52)	593 (57)
Friend	695 (64)	660 (70)	647 (45)	644 (50)	649 (55)
Stranger	722 (56)	658 (65)	669 (79)	656 (59)	653 (56)

**Table 2:** Mean d-prime and SDs (in brackets) as a function of association, priming, and bias group in Experiment 1.

	Neutral	Independent Priming		Interdependent Priming	
Associations		Low Bias	High Bias	Low Bias	High Bias
Self	2.76 (0.83)	3.32 (1.17)	3.68 (1.17)	3.35 (1.03)	3.44 (1.10)
Friend	2.19 (0.90)	2.79 (0.59)	3.18 (1.20)	2.85 (0.62)	3.17 (1.14)
Stranger	2.15 (1.10)	3.13 (0.82)	2.92 (1.01)	3.36 (1.14)	3.00 (1.21)

**Table 3:** Mean RTs and SD (in brackets) for match trials as a function of association, priming, and bias group in Experiment 2.

	Neutral	Independent Priming		Interdependent Priming	
Match RTs		Low Bias	High Bias	Low Bias	High Bias
Self	608 (64)	619 (59)	603 (57)	635 (81)	605 (59)
Friend	660 (74)	667 (75)	661 (55)	661 (79)	668 (65)
Stranger	671 (79)	666 (71)	692 (65)	667 (84)	694 (71)

**Table 4:** Mean d-prime scores and SD (in brackets) as a function of association, priming, and bias group in the Experiment 2.

		Independent Priming		Interdependent Priming	
Associations	Neutral	Low Bias	High Bias	Low Bias	High Bias
Self	3.08 (1.07)	3.20 (1.04)	3.39 (1.46)	3.24 (1.28)	3.22 (1.01)
Friend	2.55 (1.08)	2.46 (0.95)	2.74 (1.29)	2.65 (0.92)	2.58 (1.01)
Stranger	2.76 (1.42)	2.74 (1.09)	2.95 (1.25)	2.86 (0.84)	2.54 (1.12)

## Data Availability

Accession numbers will be provided during review.

## Supplementary Materials

The following supporting information can be downloaded at: https://www.mdpi.com/article/10.3390/bs12020045/s1, Table S1: Mean RTs (ms) and SDs (in brackets) for mismatched trials as a function of the shape-label association, priming and bias group in the Implicit Experiment; Table S2: Mean RTs (SD in brackets) for mismatched trails as a function of the shape-label association, priming and bias group in the Explicit Experiment; Figure S1: Decomposition of the significant interaction between priming, mismatched shape-label association and bias group using the self bias effect relative to friend (friend RT – self RT) in the mismatch trials.
