# Supplementary material for: Bicultural Minds: A Cultural Priming Approach to the Self-Bias Effect
Source: Behav Sci (Basel). 2022 Feb 11;12(2):45. doi: 10.3390/bs12020045 (PMC8869382; doi:10.3390/bs12020045)
Supplement: Supplementary file 1 [file behavsci-12-00045-s001.zip › behavsci-1563013-supplementary.pdf]

Experiment 1: Implicit priming

Neutral condition: RTs in mismatch trials

Data for the correct mismatch trials from the neutral condition were analysed using one within-subjects variable – shape-label association (self, friend, or stranger), *see Table 1*. A repeated measures ANOVA found no significant main effect of mismatched shape-label association, $F(2, 62) = 1.11, p = .34, \eta^2 = .04$.

Table S1. Mean RTs (ms) and SDs (in brackets) for mismatched trials as a function of the shape-label association, priming and bias group in the Implicit experiment.

		Independent Priming		Interdependent Priming	
Association	Neutral	Low Bias	High Bias	Low Bias	High Bias
Self	754 (63)	707 (68)	681 (67)	694 (57)	697 (57)
Friend	754 (61)	714 (56)	713 (55)	710 (47)	700 (41)
Stranger	747 (58)	706 (60)	692 (50)	697 (40)	692 (40)

Priming conditions: RTs in mismatch trials

The data from the independent and interdependent priming conditions were analysed using two within-subjects variables – priming condition (independent or interdependent) and shape-based association (self, friend, or stranger) – and a between-subjects variable – bias group (low or high bias), *see Table 1 for mean RTs*. A repeated measures ANOVA revealed a significant main effect of mismatched shape-label association, $F(2, 60) = 6.96, p < .05, \eta^2 = .19$, but not priming, $F(1, 30) = .54, p = .47, \eta^2 = .02$. Responses for mismatched self association was significantly faster than friend association ($p < .05$) but not stranger

association (p = 1.00). Stranger association was also significantly faster than friend association ($p < .01$). No interactions were found between priming and bias group, $F(1, 30) = .70$, $p = .41$, shape-label association and bias group, $F(2, 60) = .23$, $p = .79$, nor between priming and shape-label association, $F(2, 60) = 1.98$, $p = .15$. A significant three-way interaction between mismatched priming condition, shape-label association, and bias group was found, $F(2, 60) = 7.49$, $p = .001$, $\eta^2 = .20$, *see Table 1.*

The three-way interaction was decomposed by examining the self bias effect (calculated using mismatched friend RTs – self RTs) across the two priming conditions. A mixed design ANOVA revealed no significant main effect of priming, $F(1, 30) = 3.24$, $p = .08$. However, a significant interaction was found between priming (independent or interdependent) and bias group (low or high), $F(1, 30) = 12.52$, $p = .001$, $\eta^2 = .29$*, see Figure 1.*

Paired samples t-test revealed that participants in the high bias group showed significantly lower self bias relative to friend mismatch associations after interdependent priming than after independent priming, $t(15) = 3.32$, $p < .01$, $dz = .91$, *see Figure 1.* This difference was not found in participants in the low bias group, $t(15) = -1.46$, $p = .17$, $dz = .26$.

Independent samples t-test showed that the self bias relative to friend was significant smaller in the low bias group than the high bias group after independent priming, $t(30) = -2.50$, $p < .05$, $dz = .89$, *see Figure 1*, but not after interdependent priming, $t(30) = 1.03$, $p = .31$, $dz = .36$.

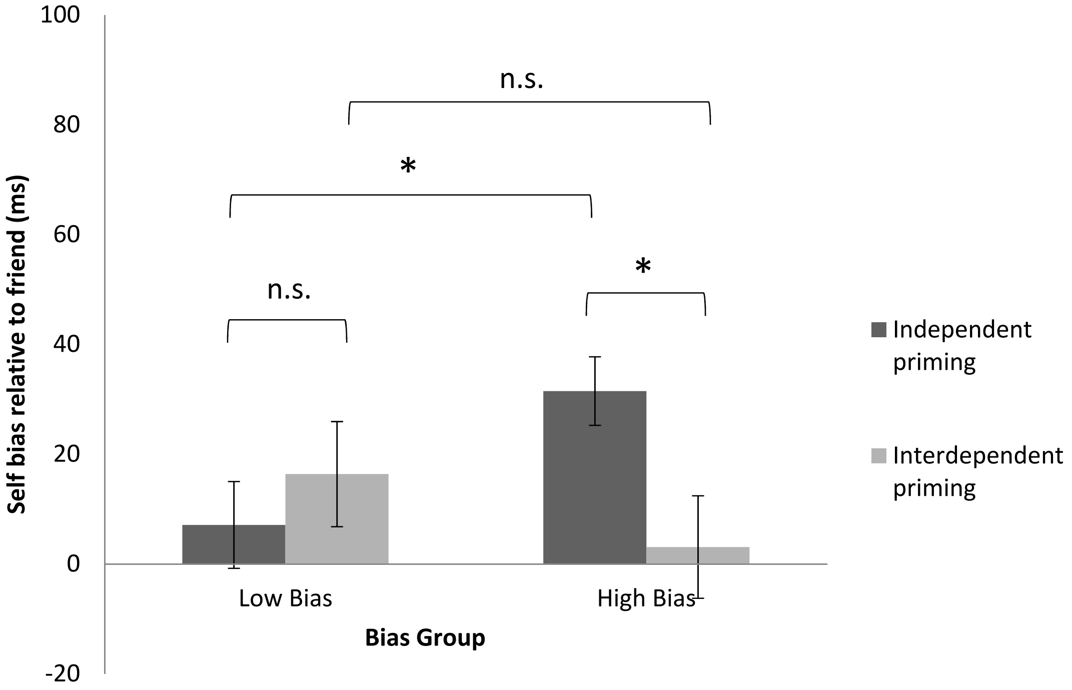

Figure S1. Decomposition of the significant interaction between priming, mismatched shape-label association and bias group using the self bias effect relative to friend (friend RT – self RT) in the mismatch trials. Mixed design ANOVA revealed a significant interaction between priming and bias group, and the paired samples t-test revealed a significant effect of priming in the high bias group. Error bars represent one standard errors. Significant results are marked with "".*

Experiment 2: Explicit priming

Neutral condition: RTs in mismatch trials

The RT data from the neutral condition on the shape-based mismatch associations were analysed using one within-subjects variable – shape-label association (friend, self, or stranger), *see Table 2*. A repeated measures ANOVA found no significant main effect of mismatched shape-label association, $F(2, 62) = .85$ $p = .43$. Response time differences were

minimal between the self and friend associations ($p = 1.00$), and self and stranger associations ($p = 1.00$). The RTs for the friend and stranger associations were also similar ($p = .62$).

Table S2. Mean RTs (SD in brackets) for mismatched trials as a function of the shape-label association, priming and bias group in the Explicit experiment.

		Independent Priming		Interdependent Priming	
Associations	Neutral	Low Bias	High Bias	Low Bias	High Bias
Self	699 (84)	701 (81)	703 (63)	703 (89)	713 (58)
Friend	701 (73)	713 (65)	703 (65)	710 (80)	719 (56)
Stranger	693 (72)	704 (77)	690 (62)	691 (74)	703 (62)

Priming conditions: RTs in mismatch trials

Data from the mismatch associations after independent and interdependent priming were analysed using two within-subjects variable – priming condition (independent or interdependent priming) and shape-based association (self, friend, or stranger) – and one between-subjects factor – bias group (low or high bias), *see Table 2.* A significant main effect of mismatched shape-label association was found, $F(2, 60) = 6.02, p < .01, \eta^2 = .17$. Responses for mismatched friend association was significantly slower than stranger association ($p < .001$). The RTs for the self association was not significantly different from friend ($p = .51$) or stranger associations ($p = .26$). No main effect of priming was found, $F(1, 30) = .14, p = .71$. No interactions were found between priming and bias group, $F(1, 30) = .69, p = .41$, nor between shape-label association and bias group, $F(2, 60) = .52, p = .60$,

nor between priming and shape-label association, $F(2, 60) = .63$, $p = .54$. No significant three-way interaction was found between priming, shape-label association and bias group, $F(2, 60) = .86$, $p = .43$.
